# Implication of intracellular localization of transcriptional repressor PLZF in thyroid neoplasms

**DOI:** 10.1186/1472-6823-14-52

**Published:** 2014-07-03

**Authors:** Kazuhiko Matsuzawa, Shoichiro Izawa, Tsuyoshi Ohkura, Hiroko Ohkura, Kiyosuke Ishiguro, Akio Yoshida, Yumi Takiyama, Masakazu Haneda, Chiaki Shigemasa, Kazuhiro Yamamoto, Shin-ichi Taniguchi

**Affiliations:** 1 Department of Regional Medicine, Tottori University Faculty of Medicine, 86 Nishi-cho, Yonago 683-8503, Japan; 2 Department of Molecular Medicine and Therapeutics, Division of Endocrinology and Metabolism, Tottori University Faculty of Medicine, 36-1 Nishi-cho, Yonago 683-8504, Japan; 3 Department of Surgery, Division of Organ Regeneration Surgery, Tottori University Faculty of Medicine, 36-1 Nishi-cho, Yonago 683-8504, Japan; 4 Division of Regenerative Medicine and Therapeutics, Institute of Regenerative Medicine and Biofunction, Tottori University Graduate School of Medical Science, 86 Nishi-cho, Yonago 683-8503, Japan; 5 Department of Medicine, Division of Metabolism and Biosystemic Science, Asahikawa Medical University, 1-1-1 Midorigaokahigashinijyo, Asahikawa 078-8510, Japan; 6 Tottori Municipal Hospital, 1-1 Matoba, Tottori 680-8501, Japan

**Keywords:** PLZF, Localization, Thyroid carcinoma, Tumourigenesis, Lymph node metastasis

## Abstract

**Background:**

Promyelocytic leukaemia zinc finger (PLZF) is a transcriptional repressor that was originally isolated from a patient with promyelocytic leukaemia. PLZF also affects key elements for cell cycle progression, such as cyclin A, and can affect the tumourigenicity of various cancers. Thus far, the behaviour of PLZF in thyroid carcinoma remains unclear.

**Methods:**

We analysed the expression profile of PLZF in different types of benign and malignant thyroid lesions as well as in normal thyroid tissue. Specifically, we examined PLZF expression in normal thyroid (N; *n =* 4), adenomatous lesion (AL; *n =* 5), follicular adenoma (FA; *n =* 2), papillary thyroid carcinoma (PTC; *n =* 20), and anaplastic thyroid carcinoma (ATC; *n =* 3) samples. PLZF expression was estimated by western blotting and immunohistochemical (IHC) staining.

**Results:**

PLZF was expressed in all samples of thyroid lesions examined. In N, AL, and FA, PLZF was mainly localized in the nucleus. In contrast, in PTC and ATC, PLZF was mainly expressed in the cytosol with high intensity. In more detail, the cytoplasmic IHC scores in PTC with capsular invasion (CI) and lymph node (LN) metastasis were higher than those in PTC without CI and LN metastasis.

**Conclusions:**

PLZF shows different subcellular localizations among PTC, ATC, and other thyroid lesions. Furthermore, high cytoplasmic expression of PLZF may be correlated with CI and LN metastasis in thyroid carcinoma. The present report is the first to describe the implications of intracellular PLZF expression in thyroid carcinomas.

## Background

Thyroid carcinoma is the most frequently occurring endocrine cancer and one of the most rapidly increasing cancers in humans [1,2]. The majority of patients with thyroid carcinoma who undergo appropriate treatment have an excellent outcome. However, in about 10% of patients, the tumour loses its ability to take up radioiodine, or becomes poorly differentiated or redifferentiated, leading to disease recurrence and death [3]. Therefore, there is a compelling need for better understanding of thyroid tumourigenesis and improved treatment for these cases.

Promyelocytic leukaemia zinc finger (PLZF), which was first identified as a partner gene fused to retinoic acid receptor alpha in the variant chromosomal translocation t(11;17)(q23;q21) in acute promyelocytic leukaemia, is a nuclear transcription factor belonging to the BTB/POZ family [4-6]. Although PLZF is mainly linked to haematological cancers, some recent studies have reported a new role for PLZF in solid cancers, such as melanomas and malignant mesothelioma [7,8]. Furthermore, wild-type PLZF has been directly linked to tumour suppression via its transcriptional repression of the c-myc oncogene [9]. These findings indicate that PLZF and PLZF derivatives can affect tumourigenesis through multiple mechanisms.

With regard to thyroid carcinoma, there have been no reports describing the possible involvement of PLZF expression and its function. We presume that PLZF in the thyroid may play a role in tumourigenesis. In the present study, we analysed the expression and intracellular distribution of PLZF in different types of benign and malignant thyroid lesions.

## Methods

### Human subjects

The study conformed to the principles outlined in the Declaration of Helsinki, and was approved by the Ethics Committee of the Faculty of Medicine, Tottori University. All patients provided written informed consent. Thyroid samples were obtained at surgery from patients as follows: normal thyroid (N; *n =* 4); adenomatous lesion (AL; *n =* 5); follicular adenoma (FA; *n =* 2); papillary thyroid carcinoma (PTC; *n =* 20); anaplastic thyroid carcinoma (ATC; *n =* 3). We performed immunohistochemical (IHC) staining in all samples, whereas western blot analyses were carried out using four samples of N (cases 1–4), five samples of AL (cases 5–9), and 13 samples of PTC (cases 12–24). We performed IHC staining using four samples of N, five samples of AL, two samples of FA, 20 samples of PTC, and three samples of ATC. The characteristics of the analysed samples are listed in Table 1.

**Table 1:** Summary of the thyroid neoplasm histotypes evaluated and the patient sex and age distributions

**Histotypes**	**n**	**Age**	**Sex (M/F)**
Normal thyroid	4	67 ± 15	2/2
Adenomatous lesion	5	43 ± 11	0/5
Follicular adenoma	2	32 ± 9	0/2
Papillary carcinoma	20	57 ± 16	4/16
CI(-), LN meta(-)	5	51 ± 23	2/3
CI(+), LN meta(-)	8	64 ± 13	0/8
CI(+), LN meta(+)	7	51 ± 13	2/5
Anaplastic carcinoma	3	68 ± 9	2/1

CI; capsular invasion, LN meta; lymphnode metastasis.

### Western blot analysis

Human thyroid tissues were homogenized in 300 μl of RIPA buffer (1% Nonidet P-40, 0.5% sodium deoxycholate, and 0.1% sodium dodecyl sulphate in PBS with a protease inhibitor cocktail (Roche Molecular Biochemicals, Indianapolis, IN)) and centrifuged at 15000 rpm for 20 min at 4°C. The supernatants were used as the total cell lysates. A aliquot (10 μg protein) of each lysate was subjected to 10% SDS-PAGE, and the separated proteins were electrophoretically transferred to nitrocellulose membranes. The membranes were blocked with Tris-buffered saline containing 0.05% Tween-20 and 5% non-fat dried milk, washed, and incubated with polyclonal primary antibodies against PLZF (sc-22839; Santa Cruz Biotechnology Inc., Santa Cruz, CA; 1:200 dilution) and β-actin (sc-1616; Santa Cruz Biotechnology Inc.; 1:500 dilution). The membranes were then exposed to an anti-rabbit secondary antibody (NA934; Amersham Pharmacia Biotech, Little Chalfont, UK; 1:5000 dilution). After incubation with the secondary antibody, detection of the bound antibodies was performed using enhanced chemiluminescence.

### IHC evaluation of PLZF expression

PLZF expression was analysed by IHC staining of thyroid samples. Paraffin-embedded tissue sections (4-μm thickness) were deparaffinized in xylene and rehydrated through a graded alcohol series to deionized water. After microwave antigen retrieval, the endogenous peroxidase activity was blocked with H_2_O_2_. The sections were then sequentially incubated with the anti-PLZF primary antibody (1:50 dilution) for 12 h at 4°C, and a peroxidase-labelled anti-rabbit secondary antibody (414141 F; Nichirei, Tokyo, Japan) for 30 min at room temperature. After colour development with 3,3′-diaminobenzidine and counterstaining with haematoxylin, the sections were scanned at various magnifications (×100 to × 400) using light microscopy.

The proportions of positively stained cells and the intensity scores were evaluated by two experienced professionals. The Allred scoring system [10] was used for PLZF staining interpretation. The proportion of positively stained cells was rated as follows: 0%, no positive staining; 1, between 0% and 1% positive staining; 2, between 1% and 10% positive staining; 3, between 10% and 33% positive staining; 4, between 33% and 66% positive staining; 5, between 66% and 100% positive staining. In addition to the proportion score, an intensity score was made on the basis of the average intensity of staining as follows: 0, negative; 1, weak; 2, intermediate; 3, strong. The intensity score and proportion score were added to obtain the total score, termed the IHC score, which is referred to as the Allred score [10] and is either 0 or between 2 and 8.

### Statistical analysis

The western blotting values by densitometric analysis and IHC scores for the individual samples were compared by the Tukey’ HSD test. The IHC scores were compared between the nucleus and cytoplasm, and between positivity or negativity for capsular invasion (CI) and lymph node (LN) metastasis in PTC using the Mann–Whitney U test. Values of *p <* 0.05 were considered to indicate statistical significance. All statistical analyses were performed using SPSS 14.0 for Windows (SPSS Corp., Tokyo, Japan).

## Results

### Expression of PLZF in human thyroid samples

**Figure 1:**
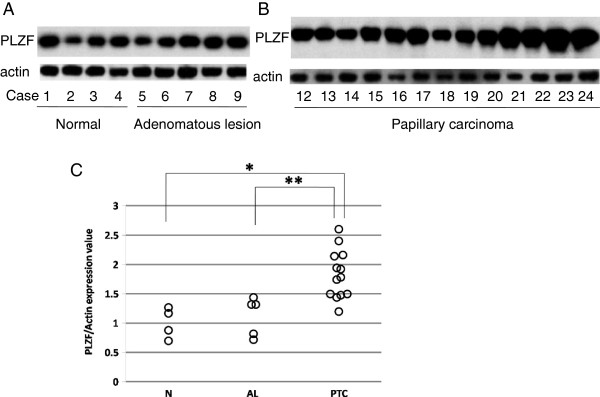
**Western blot analyses of PLZF in normal thyroid, adenomatous lesion (A), and papillary thyroid carcinoma (B) samples.** For immunoblotting, an aliquot (10 μg protein) of each lysate was loaded. To compare the PLZF expression levels among different membranes, we used the PLZF/β-actin expression ratios, representing the densitometrically analysed data for each membrane divided by the mean value for normal thyroid tissues, which was used as an internal control **(C)**. *p < 0.05, vs. normal thyroid and papillary thyroid carcinoma; **p < 0.05, vs. adenomatous lesion and papillary thyroid carcinoma.

We estimated PLZF expression by western blot analyses. All samples were confirmed to express PLZF (Figure 1A and B). All samples of PTC showed high expression of PLZF (Figure 1B). Conversely, the PLZF expression levels were relatively low in N and AL compared with PTC (Figure 1A). To compare the PLZF expression levels between different membranes, we used the PLZF expression ratios, representing the densitometrically analysed data for each membrane divided by the mean value for N, which was used as an internal control (Figure 1C). The PLZF/actin expression ratio was significantly higher in PTC than in both N and AL (*p <* 0.05).

### Localization of PLZF in different types of benign and malignant thyroid lesions

**Figure 2:**
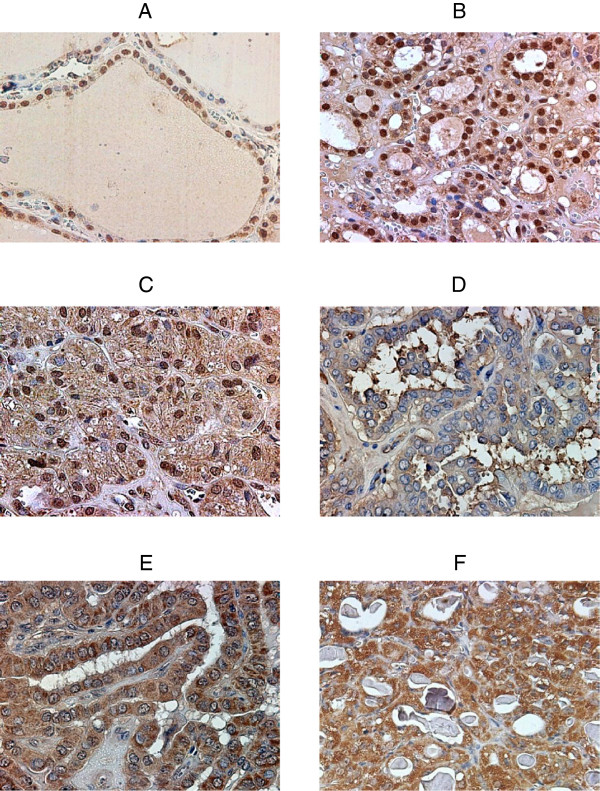
**PLZF expression in a normal thyroid, an adenomatous lesion, a follicular adenoma, two papillary thyroid carcinomas with different degrees of capsular invasion and lymph node metastasis, and an anaplastic thyroid carcinoma representative of our study population.** All images are × 400 magnification. IHC: immunohistochemical; N/C: nucleus/cytoplasm. **(A)** Normal thyroid showing nuclear staining (IHC score: N/C = 7/2). **(B)** Adenomatous lesion showing strong nuclear staining with intermediate cytoplasmic staining (IHC score: N/C = 7/3). **(C)** Follicular adenoma showing strong nuclear staining with intermediate cytoplasmic staining (IHC score: N/C = 7/4). **(D)** Papillary thyroid carcinoma without capsular invasion and lymph node metastasis showing faint cytoplasmic staining (IHC score: N/C = 0/3). **(E)** Papillary thyroid carcinoma with both capsular invasion and lymph node metastasis showing strong cytoplasmic staining (IHC score: N/C = 0/8). **(F)** Anaplastic thyroid carcinoma showing strong cytoplasmic staining (IHC score: N/C = 0/8).

**Figure 3:**
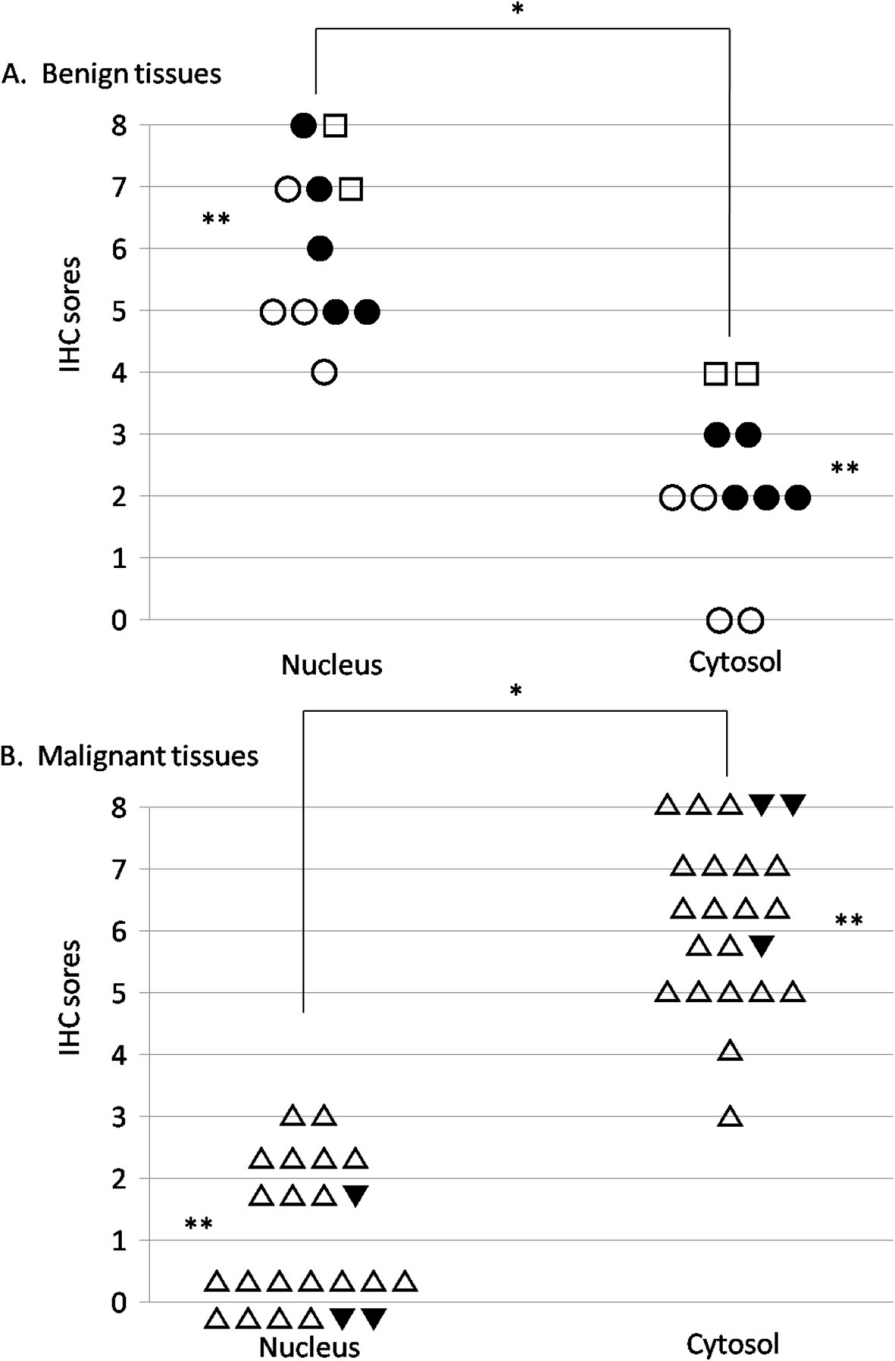
**Comparison of PLZF expression between the nucleus and cytosol of benign and malignant tumours. (A, B)** Differences in PLZF expression between the nucleus and cytosol of benign tissues **(A)** and malignant tissues **(B)**. *p < 0.05, nucleus vs. cytosol; **p < 0.05, benign tissues vs. malignant tissues. Open circle: normal thyroid; filled circle: adenomatous lesion; open square: follicular adenoma; open triangle: papillary thyroid carcinoma; filled triangle: anaplastic thyroid carcinoma.

**Figure 4:**
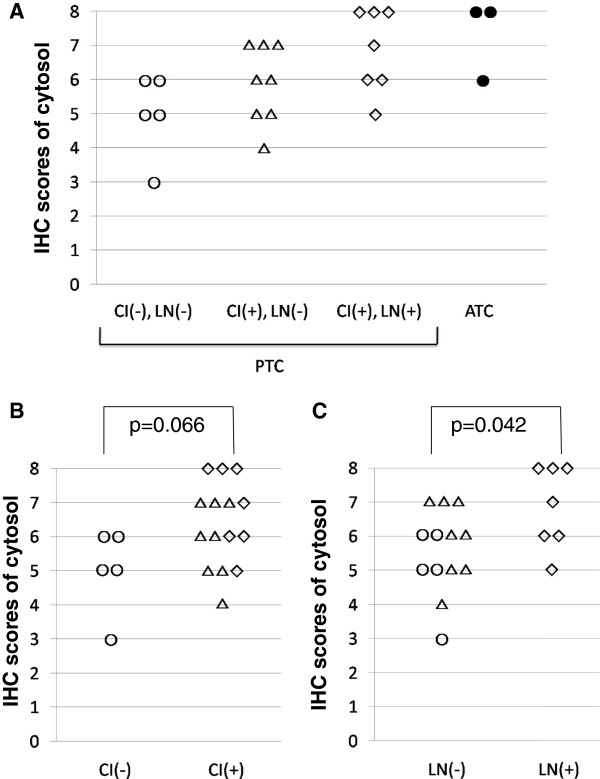
**Comparison of PLZF expression in the cytoplasm among papillary thyroid carcinomas and anaplastic thyroid carcinomas. (A)** The papillary thyroid carcinoma samples were classified according to capsular invasion and lymph node metastasis. **(B, C)** Differences in cytosolic PLZF expression between capsular invasion-positive and capsular invasion-negative tumours **(B)** and between lymph node metastasis-positive and lymph node metastasis-negative tumours **(C)**. Open circle: papillary thyroid carcinoma without capsular invasion and lymph node metastasis; open triangle: papillary thyroid carcinoma with capsular invasion; white diamond: papillary thyroid carcinoma with lymph node metastasis; filled circle: anaplastic thyroid carcinoma.

We estimated the intracellular distribution of PLZF by IHC staining. In N (Figure 2A), PLZF was mainly localized in the nucleus. The strength of its expression ranged from intermediate to strong. However, the expression of PLZF in the cytosol was faint or absent. The IHC scores were 4–7 in the nucleus and 0 or 2 in the cytosol. In AL (Figure 2B), PLZF was mainly localized in the nucleus, and its strength was intermediate or strong. In the cytosol, the PLZF expression was weak, but slightly stronger than in N. The IHC scores were 5–8 in the nucleus and 2 or 3 in the cytosol. In FA (Figure 2C), PLZF was mainly localized in the nucleus. In the cytosol, the PLZF expression was stronger than in N. The IHC scores were 7 or 8 in the nucleus and 4 in the cytosol.In almost all samples of PTC, the PLZF expression in the nucleus was absent or very weak. In general, the PLZF expression was strong in the cytosol. However, the proportion of positive cells and the intensity of staining varied according to each case. As shown in Figure 2D, the intensity of PLZF staining was very weak or absent and the proportion of positive cells was low. Conversely, as shown in Figure 2E, the intensity of PLZF staining was strong and the proportion was high, despite the same histotype (PTC). In all samples of ATC (Figure 2F), PLZF was localized in the cytosol and the intensity of staining was high, resembling the findings shown in Figure 2E.We clarified the staining patterns of PLZF between benign and malignant thyroid lesions by grading using the IHC scores. In benign lesions, such as N, AL, and FA, PLZF was localized in the nucleus (Figure 3A). In contrast, in malignant lesions, such as PTC and ATC, PLZF was localized in the cytosol (Figure 3B). There was a significant difference between benign and malignant thyroid lesions.Subsequently, we examined PTC in more detail because there were different proportions and intensities of IHC staining in the cytosol. We classified PTC according to CI and LN metastasis. As shown in Figure 4A, the IHC scores tended to gradually increase from CI(-)LN metastasis(-) to CI(+)LN metastasis(-), CI(+)LN metastasis(+), and ATC, albeit without statistical significance. The IHC scores in samples with CI were higher than those in samples without CI (Figure 4B), although the difference was not significant. With respect to LN metastasis, the PLZF expression levels in samples with LN metastasis were significantly higher than those in samples without LN metastasis (Figure 4C).

## Discussion

PLZF expression has been detected in most human tissues [11]. However, its expression in the thyroid has not been reported. By western blot analyses, we were able to confirm PLZF expression in N, AL, and PTC, and showed that PLZF was expressed at quite high levels in PTC compared with N and AL. PLZF is known to be a transcriptional repressor, and is associated with suppression of cellular proliferation. In a variety of cell models, sustained PLZF expression was associated with cell cycle arrest in G1 and eventual apoptosis [12-14]. Therefore, we presumed that the PLZF expression in benign thyroid samples would be higher than that in malignant samples. However, our results indicated alternative expression levels. In a previous report on myeloid cell lines, cytokines were shown to mediate inactivation of PLZF by triggering its export from the nucleus in an ERK-dependent manner, leading to interference with PLZF-mediated repression of growth and differentiation [15]. In this manner, PLZF was required to be localized in the nucleus to perform its functional roles. Therefore, the intracellular localization of PLZF would be as important as the amount of its expression.

To investigate the localization of PLZF, we performed IHC staining in different types of benign and malignant thyroid lesions as well as in normal thyroid tissue. Our findings revealed a significant difference in PLZF localization between benign and malignant thyroid lesions. As one of the mechanisms for PLZF intracellular translocation, Nanba et al. [16] reported that heparin-binding EGF-like growth factor (HB-EGF) generated by ectodomain shedding of proHB-EGF causes nuclear export of PLZF, increases the expression of cyclin A, and promotes S-phase entry. Furthermore, an interaction between the HB-EGF carboxy-terminal region (HB-EGF-C) and PLZF occurs in a mouse skin model of keratinocyte hyperplasia. Ota et al. [17] reported that in clinical IHC study, increased expression of HB-EGF was observed in thyroid carcinoma samples more than benign thyroid samples. Although, we could not clarify an interaction between PLZF and HB-EGF-C in the thyroid, it is reasonable to presume that the similar mechanism involving HB-EGF-C may lead to the translocation of PLZF in PTC and APC.

When we observed the IHC scores for PTC in more detail, the cytoplasmic IHC scores in PTC with CI and LN metastasis were higher than those in PTC without CI and LN metastasis. These findings indicate that the strength of PLZF expression in the cytosol reflects the progression of malignancy, such as CI and LN metastasis. A previous report suggested that down-regulation of microRNA-126 and -126* may be a key event in melanoma progression [18]. These microRNAs play a tumour suppressor role in human melanoma through down-modulation of two metalloproteinases, ADAM9 and MMP7, resulting in decreased HB-EGF activation [18]. This mechanism was thought to eventually bring about export of PLZF from the nucleus to the cytosol. If this is the case, intracellular translocation of PLZF would be correlated with melanoma progression. Although we were unable to clarify whether the same mechanism acts in the thyroid, our findings suggest a hypothesis that the amount of PLZF in the cytoplasm may be one of the possible causes of the progression of thyroid carcinoma.

The interaction between HB-EGF-C and PLZF is considered to be a novel therapeutic target. In a previous study, Telmisartan was found to block the binding of HB-EGF-C to PLZF, and inhibit the cell proliferation of human colon cancer cell lines [19]. Replacement of microRNA-126 and -126* might be considered a promising therapeutic approach against malignant melanoma [18]. In this study, we could not clarify the reason why PLZF is translocated to the cytoplasm and highly expressed in the cytosol during PTC progression. Therefore, further studies are necessary to reveal the underlying mechanism. If a similar mechanism to colon cancer and malignant melanoma exists for thyroid carcinoma, PLZF localization may be a novel therapeutic target to suppress the progression of thyroid carcinoma.

## Conclusion

In the present study, we have demonstrated four important findings about PLZF in thyroid samples. First, we confirmed that PLZF is expressed in the thyroid. Second, the levels of PLZF expression were greater in PTC than in N and AL. Third, the intracellular localization of PLZF in PTC and ATC showed a different pattern compared with benign thyroid lesions. Finally, as thyroid carcinoma progressed through CI, LN metastasis, and ATC, the PLZF expression became higher. To our knowledge, this is the first report to describe the expression and localization of PLZF in the thyroid. The present results suggested that the cytosolic localization and intensity of PLZF expression may be correlated with thyroid tumourigenicity and degree of malignancy.

## Abbreviations

PLZF: Promyelocytic leukaemia zinc finger; N: Normal thyroid; AL: Adenomatous lesion; FA: Follicular adenoma; PTC: Papillary thyroid carcinoma; ATC: Anaplastic thyroid carcinoma; IHC: Immunohistochemical; CI: Capsular invasion; LN: Lymph node; HB-EGF: Heparin-binding EGF-like growth factor; HB-EGF-C: Heparin-binding EGF-like growth factor carboxy-terminal region.

## Competing interests

The authors declare that they have no competing interests.

## Authors’ contributions

KM participated in the design of the study, carried out molecular studies, and performed the statistical analyses. KM and YT carried out immunohistochemical staining. KI collected the data. ST, and CS conceived the study. MH, ST, KY, AY, HO, TO, and SI participated in the study design and coordination, and helped to draft the manuscript. All authors read and approved the final manuscript.

## Pre-publication history

The pre-publication history for this paper can be accessed here:

http://www.biomedcentral.com/1472-6823/14/52/prepub
